# Making Science Computable Using Evidence-Based Medicine on Fast Healthcare Interoperability Resources: Standards Development Project

**DOI:** 10.2196/54265

**Published:** 2024-06-25

**Authors:** Andrey Soares, Lisa M Schilling, Joshua Richardson, Bhagvan Kommadi, Vignesh Subbian, Joanne Dehnbostel, Khalid Shahin, Karen A Robinson, Muhammad Afzal, Harold P Lehmann, Ilkka Kunnamo, Brian S Alper

**Affiliations:** 1 Department of Medicine University of Colorado Anschutz Medical Campus Aurora, CO United States; 2 Center for Informatics Research Triangle Institute International Berkeley, CA United States; 3 Quantica Computacao Hyderabad India; 4 Scientific Knowledge Accelerator Foundation Franklin, NC United States; 5 College of Public Health, Department of Epidemiology and Biostatistics University of Arizona Tucson, AZ United States; 6 Computable Publishing LLC Franklin, NC United States; 7 Department of Medicine Johns Hopkins School of Medicine Baltimore, MD United States; 8 Department of Computing and Data Science Birmingham City University England United Kingdom; 9 Duodecim Publishing Company Ltd Helsinki Finland

**Keywords:** evidence-based medicine, FHIR, Fast Healthcare Interoperability Resources, computable evidence, EBMonFHIR, evidence-based medicine on Fast Healthcare Interoperability Resources

## Abstract

**Background:**

Evidence-based medicine (EBM) has the potential to improve health outcomes, but EBM has not been widely integrated into the systems used for research or clinical decision-making. There has not been a scalable and reusable computer-readable standard for distributing research results and synthesized evidence among creators, implementers, and the ultimate users of that evidence. Evidence that is more rapidly updated, synthesized, disseminated, and implemented would improve both the delivery of EBM and evidence-based health care policy.

**Objective:**

This study aimed to introduce the EBM on Fast Healthcare Interoperability Resources (FHIR) project (EBMonFHIR), which is extending the methods and infrastructure of Health Level Seven (HL7) FHIR to provide an interoperability standard for the electronic exchange of health-related scientific knowledge.

**Methods:**

As an ongoing process, the project creates and refines FHIR resources to represent evidence from clinical studies and syntheses of those studies and develops tools to assist with the creation and visualization of FHIR resources.

**Results:**

The EBMonFHIR project created FHIR resources (ie, *ArtifactAssessment*, *Citation*, *Evidence*, *EvidenceReport*, and *EvidenceVariable*) for representing evidence. The COVID-19 Knowledge Accelerator (COKA) project, now Health Evidence Knowledge Accelerator (HEvKA), took this work further and created FHIR resources that express *EvidenceReport*, *Citation*, and *ArtifactAssessment* concepts. The group is (1) continually refining FHIR resources to support the representation of EBM; (2) developing controlled terminology related to EBM (ie, study design, statistic type, statistical model, and risk of bias); and (3) developing tools to facilitate the visualization and data entry of EBM information into FHIR resources, including human-readable interfaces and JSON viewers.

**Conclusions:**

EBMonFHIR resources in conjunction with other FHIR resources can support relaying EBM components in a manner that is interoperable and consumable by downstream tools and health information technology systems to support the users of evidence.

## Introduction

### Background and Significance

Timely and relevant biomedical evidence is essential to provide high-quality health care. Decision makers rely on “synthesized evidence” from systematic reviews (SRs) and clinical practice guidelines (CPGs) to inform clinical care decisions and policies at multiple levels [1-7]. Therefore, actionable evidence is key for optimizing health care delivery and outcomes. Yet, with an estimated 75 clinical trials and 11 SRs published every day [8], efforts to synthesize, disseminate, and implement biomedical evidence throughout the evidence ecosystem to inform decision-making are unsustainable [9].

Evidence-based medicine (EBM) is increasingly recognized as critical in the decision-making process related to patient care and policy development. EBM is “the conscientious, explicit, and judicious use of current best evidence in making decisions about the care of individual patients” [10]. The practice of EBM depends on comprehensive and up-to-date synthesized research evidence, which requires continuous updating and reconciliation of new scientific results with previous results. For example, early trial results on prenatal steroids for preterm births were initially inconclusive, but evidence syntheses justified steroidal therapy as the best practice [11-14]. However, EBM practice is laborious, methodologically complex, and often error prone.

Multiple barriers hinder the effective and efficient use of evidence syntheses. First, the time and effort required to synthesize evidence often leads to SRs and CPGs being out of date by the time of their publication [15,16]. Even when synthesized evidence is available in digital form, it is typically in narrative form and accessed via a bibliographical database that requires proactive searching (eg, PubMed or a publisher), an email newsletter (eg, *Journal of the American Medical Association Internal Medicine Newsletter*), or social media notification (eg, X, formerly known as Twitter, or Doximity). These “pull” methods of synthesized evidence are helpful means of dissemination, but they do not facilitate more efficient “push” methods that promote use, implementation, and action at scale. Second, the common mode of evidence dissemination, which is human-readable text, is not standardized in ways that support technical solutions to scalable dissemination and implementation [15-19]. Despite notable past efforts for structuring and disseminating guidance, such as the GuideLine Interchange Format (GLIF) [20,21] and Standards-Based Sharable Active Guideline Environment (SAGE) [22] standards, there are no existing scalable and reusable computer-readable standards for distributing research results and synthesized evidence among creators, implementers, and the ultimate users of that evidence.

The current state is an ecosystem of researchers, informaticians, statisticians, policy makers, epidemiologists, librarians, and other stakeholders in the biomedical research community who synthesize evidence by manually searching for relevant studies, assessing the studies for quality and risk of bias, and compiling the results in labor-intensive ways. Evidence implementers, including CPG and clinical decision support (CDS) creators, whether they are creating or implementing local or third-party tools, must similarly review and verify the evidence before use. The challenges with finding and delivering evidence are amplified by needs due to the COVID-19 pandemic [23-28], where “scientists [have] published well over 100,000 articles about the coronavirus pandemic in 2020” [29] and for which there are currently over 2.5 million publications [30]. Today, these mostly manual, redundant, and disjointed processes seem to be the acknowledged status quo, even though new evidence continues to be generated at a rapid pace. The evidence synthesis ecosystem is, therefore, rife with duplication and uncoordinated efforts [31,32] for identifying, appraising, synthesizing, and disseminating evidence, requiring considerable resources, expertise, and time [33,34]. Evidence that is more rapidly updated, synthesized, disseminated, and implementable would improve both the delivery of EBM and evidence-based health care policy [4,35].

### Computable Evidence

**Figure 1:**
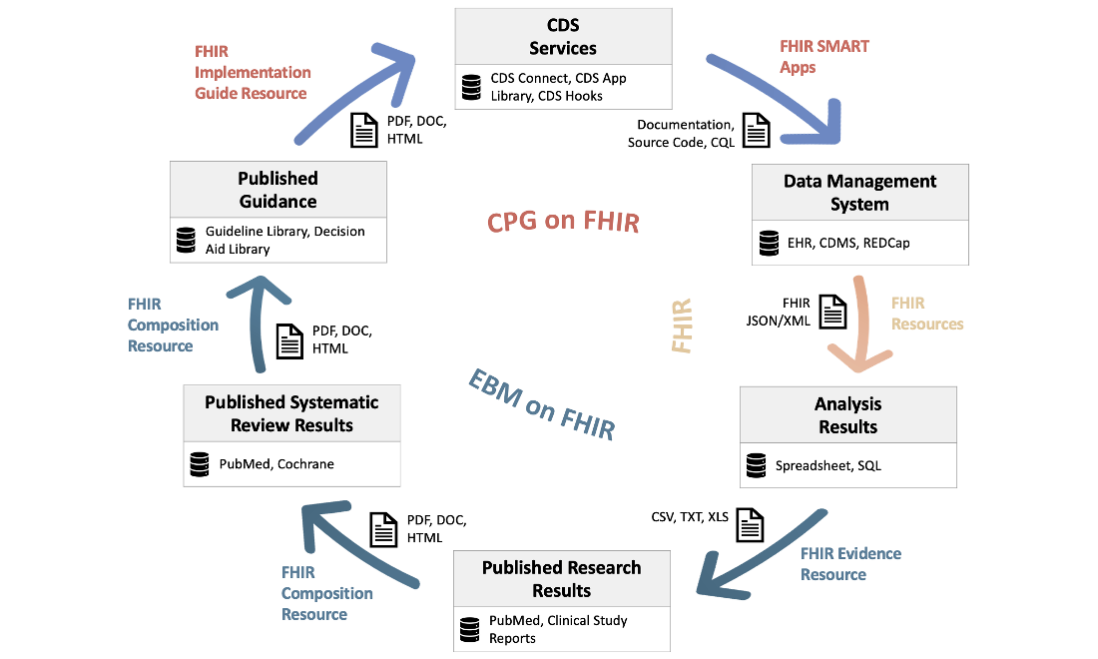
Application of Fast Healthcare Interoperability Resources (FHIR) resources to the evidence ecosystem. EBMonFHIR denotes application to results of scientific analysis, synthesis, and publication. CPGonFHIR denotes application to guidance and decision support. CSV, TXT, XLS, PDF, DOC, HTML, JSON, and XML denote file formats. CDMS: clinical data management system; CDS: clinical decision support; CPG: clinical practice guideline; CQL: Clinical Quality Language; EBM: evidence-based medicine; EBMonFHIR: Evidence-Based Medicine on Fast Healthcare Interoperability Resources; EHR: electronic health record; REDCap: Research Electronic Data Capture. Adapted from the “Digital and Trustworthy Evidence Ecosystem” [38].

Solutions to improve the evidence-to-practice lifecycle [36] should begin with the transformation of study results into “computable evidence” or “knowledge artifacts” that could be consumed by software-based information systems. Key to computable evidence is knowledge representation in machine-interpretable formats that enable findable, accessible, interoperable, and reusable [37] information across the evidence ecosystem. Figure 1 (adapted from the “Digital and Trustworthy Evidence Ecosystem” [38]) depicts this vision where research results at many stages throughout analysis, publication, and synthesis (the EBM on Fast Healthcare Interoperability Resources [EBMonFHIR] area) can be extracted and transformed from numerous disparate sources (such as registry reports, gray literature, preprints, and peer-reviewed literature databases) and stored as machine-readable evidence in an interoperable standard format that could then be used by a wide variety of evidence users and developers from biomedical knowledge bases (ie, evidence repositories) to SRs, CPGs, and CDS systems. Figure 1 also shows where related interoperability solutions [39] applied to CPG and CDS representation (the CPGonFHIR area), such as CDS Connect (a platform with a repository and authoring tool for CDS artifacts) [40] and CDS Hooks (a Health Level Seven [HL7] specification that provides a way to embed CDS services within the clinician workflow of an electronic health record) [41], can complete the evidence-to-practice implementation within the evidence ecosystem.

### The EBMonFHIR Project

Built upon the success of Fast Healthcare Interoperability Resources (FHIR) [42-44] to promote interoperability and standards for data exchange, we initiated the EBMonFHIR project on May 16, 2018. The aim was to extend the methods and infrastructure of HL7 FHIR to provide an interoperability standard for the electronic exchange of biomedical knowledge from and about clinical research and recommendations [45]. We sought to stand on the shoulders of those who have previously made strides in structuring evidence and standardizing the means to share that evidence (eg, GLIF) by leveraging FHIR’s growing popularity and acceptance. The project solicits the participation of known experts and gathers input from broad communities in the evidence ecosystem to determine the data exchange needs for interoperable knowledge assets [45] and defines the FHIR resources related to the domain of clinical research evidence. We have begun showing that FHIR can deliver not just clinical data but also synthesized evidence and knowledge to end users.

### Historical Context

The project started with implementers from EBSCO Health, Duodecim Medical Publications Ltd, HarmoniQ, and MAGIC Evidence Ecosystem Foundation and expanded to a multisector project with participation from academia, industry, government, and nonprofit organizations [46].

The COVID-19 pandemic introduced a new impetus in the quest to make biomedical evidence computable and interoperable. In light of the pandemic, where scientists and clinicians urgently needed timely results and evidence, the EBMonFHIR project leaders organized a larger group named the “COVID-19 Knowledge Accelerator (COKA) Initiative” and shifted their focus to the biomedical evidence regarding SARS-CoV-2 [47]. COKA is a nonprofit unincorporated organization with global collaboration to develop and advance interoperability standards for COVID-19 knowledge and to enhance the evidence exchange standards. As of January 2023, the COKA initiative was renamed to Health Evidence Knowledge Accelerator (HEvKA) to better represent the group’s now broader scope that is inclusive of clinical, public health, and environmental health domains.

### Objectives

In this paper, we introduce the EBMonFHIR project that aims to produce an HL7 FHIR schema to express biomedical evidence as computable evidence—as well as FHIR resource instances, terminologies, and tools—and to promote an effective and efficient evidence ecosystem. We describe the participants involved in this effort, the process to develop the EBMonFHIR standards, and the progress made to represent evidence findings as FHIR resources. We also provide information on initial impacts, limitations, and next steps for stakeholders involved in standards and controlled terminology development, EBM implementation, and evidence used for both clinical studies and syntheses of those studies. We finally describe how others can get involved in the EBMonFHIR effort by way of the HEvKA initiative.

## Methods

HEvKA is advancing EBMonFHIR according to the five aspects of the HL7 standards development process [48], and they are (1) foster consensus, (2) ensure content is fit for purpose, (3) ensure content is implementable, (4) establish an appropriate implementer community, and (5) ensure ongoing maintenance of the standard. Through up to 15 online meetings each week and up to 3 HL7 FHIR Connectathons [49] each year, HEvKA creates and refines FHIR resources to represent evidence from clinical studies and syntheses of those studies and develops tools to assist with the creation and visualization of FHIR resources.

Updates and improvements for any FHIR specification can be developed, proposed, reviewed, improved, voted on, and released within a documented environment in accordance with the American National Standards Institute (ANSI)-sanctioned HL7 ballot process, and ultimately published as part of the official HL7 FHIR specification. [50]

The FHIR resources that HEvKA develops are continuously revised and adapted to reflect the best representation of the knowledge from the community. The HL7 CDS Work Group [51] is responsible for evaluating and approving the additions and changes to these FHIR resources.

To support a culture of transparency and openness about the process of developing the FHIR resources and support tools, HEvKA makes use of numerous digital approaches to document and keep track of its activities and disseminate its progress, including the HL7 Confluence Web Page (content management) [45], Google Drive (content repository) [52], and Microsoft Teams (videoconference) [53]. The content produced is open-source and freely available to the community, with examples published on the Fast Evidence Interoperability Resources (FEvIR) Platform [54]. HEvKA also coordinates input and dissemination across many communities in the evidence ecosystem.

HEvKA has 14 active working groups that meet weekly to address different aspects of the project (ie, Communications; Computable EBM Tools Development; CQL Development; EBM Implementation Guide; Eligibility Criteria; Funding the Ecosystem Infrastructure; GRADE [Grading of Recommendations Assessment, Development and Evaluation] Ontology; Measuring the Rate of Scientific Knowledge Transfer; Ontology Management; Project Management; Risk of Bias Terminology; Setting the Scientific Record on FHIR; Statistic Terminology; and StatisticsOnFHIR). For details, visit the Confluence web page for information about HEvKA [55].

## Results

### EBM Representation With FHIR Resources

HEvKA first created FHIR resources for representing research results (*Evidence*) and variable definitions (*EvidenceVariable*); and after March 2020, HEvKA further created FHIR resources that express compositions (*EvidenceReport*), citations (*Citation*), and judgments about knowledge (*ArtifactAssessment*). To ease the readability of this article, we will broadly refer to these FHIR resources as the “EBMonFHIR Resources.” Table 1 shows the list of EBMonFHIR resources the project developed. From here on, we list FHIR resources, FHIR elements, and data types [56] in *italics*.

An *Evidence* resource [57] provides an expression of the most granular components of evidence. Evidence is often represented by values and parameters of statistical measures (eg, mean, confidence interval, relative risk, and hazard ratio) and expressions of certainty or classifications of these statistical findings. These statistics are about a particular combination of variables from a particular study, so the *Evidence* resource can refer to *EvidenceVariable* resources for definitions of the observed or intended variables. However, to support interoperability across systems, the *Group* resource [58] may be used instead of an *EvidenceVariable* resource [59], especially when referring to a group of people such as a population, sample, or subgroup.

**Figure 2:**
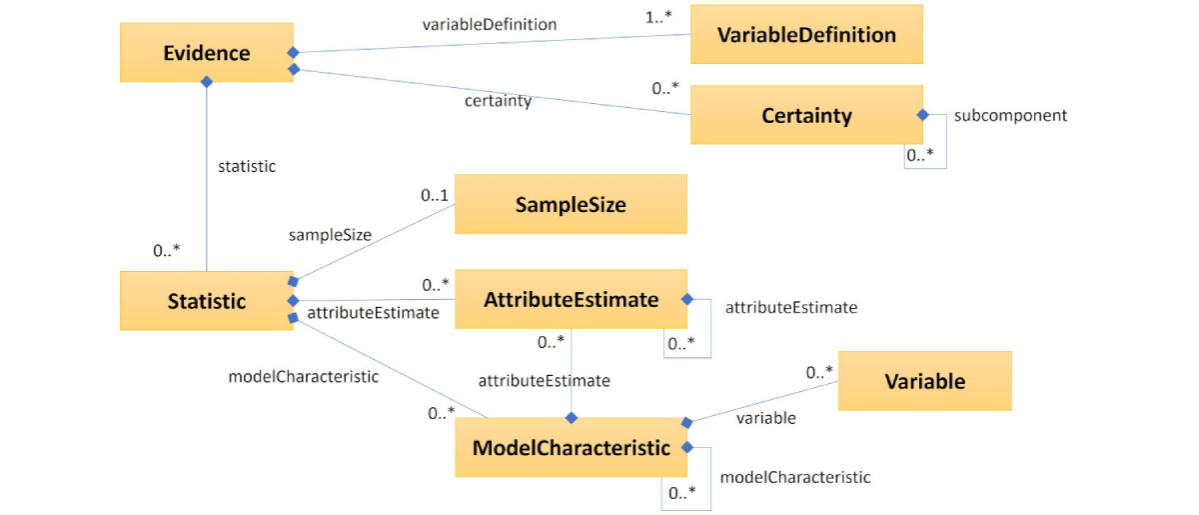
*Evidence* resource backbone elements are represented with an abbreviated unified modeling language diagram. 0..1 denotes the element is optional and can only have 1 instance. 0..* denotes the element is optional and can have any number of instances. 1..* denotes the element is required and can have any number of instances.

The *Evidence* resource refers to statistical measures and their values with a machine-interpretable expression of a statistic, including the quantity; unit of measure; classification of statistic type; sample size; attribute estimates such as confidence intervals, *P* values, and heterogeneity estimates; and statistical model characteristics (Figure 2). The *statisticType* element has, as of November 2, 2023, a suggested set of 22 possible codes [60], assembled by the HEvKA team, that represent types of statistics (eg, median, relative risk, and incidence rate ratio). HEvKA has drafted 139 terms for statistic types in total (these will eventually replace the Statistic Type Value Set).

Biomedical evidence is comprised of facts and interpretations derived from an analysis of observations of a selective sample. Certainty about any evidence may change due to methodological factors, statistical factors, and contextual factors, and for the relatedness between the sample, the evidence was derived from the population to which the evidence is applied. The *certainty* element provides a machine-interpretable expression of confidence in, or certainty or quality of, the evidence. The *type* subelement can express the aspect of the certainty being rated, using codable concepts from a suggested value set (eg, including the overall certainty, risk of bias, inconsistency, indirectness, imprecision, publication bias, dose-response gradient, plausible confounding, and large effect size [61]).

An *EvidenceVariable* resource [59] provides an expression of a single evidence variable (eg, a single exposure or a single outcome or measured variable).

The *Citation* resource “enables reference to any knowledge artifact for purposes of identification and attribution” [62], including “location, authorship, and contributorship to a journal article, report, document, resource, or other knowledge artifact” [62]. For instance, the *Citation* resource [63] is a reference to the *Evidence* resource [64], representing the primary outcome from an article reporting the results of a randomized clinical trial [65].

The *EvidenceReport* resource represents a “container for a collection of resources and codable concepts, adapted to support compositions of *Evidence*, *EvidenceVariable*, and *Citation* resources and related concepts” [66]. This resource may bundle knowledge from 1 or multiple studies [66]. The report can be represented in sections of different forms including text, references to codable concepts, or other FHIR resources and sections. The *EvidenceReport* resource is “suited for communicating reports about research and data analysis not specific to individual persons” [66], distinct from the FHIR *Composition* resource commonly used for reports specific to individual persons. However, the *EvidenceReport* resource will be deprecated as the *Composition* resource has been modified to include an EvidenceReport profile to support EBMonFHIR use.

The *ArtifactAssessment* resource “represents one or more assessments of another record or resource” [67]. This resource covers assessments about clinical records, health care provision, and records related to community knowledge (eg, evidence), and may include comments, corrections, classifications, ratings, questions, and responses.

**Table 1:** List of FHIR^a^ resources developed by the EBMonFHIR^b^ and HEvKA^c^ projects (FHIR version 6.0.0 current build, as of November 2, 2023).

FHIR^a^ resource	Description	Reference to URL
*ArtifactAssessment*	The *ArtifactAssessment* resource provides 1 or more comments, classifiers, or ratings about a resource and supports attribution and rights management metadata for the added content.	[67]
*Citation*	The *Citation* resource enables reference to any knowledge artifact for purposes of identification and attribution. The *Citation* resource supports existing reference structures and developing publication practices such as versioning, expressing complex contributorship roles, and referencing computable resources.	[62]
*Evidence*	The *Evidence* resource provides a machine-interpretable expression of an evidence concept including the evidence variables (eg, population, exposures or interventions, comparators, outcomes, measured variables, confounding variables), the statistics, and the certainty of this evidence.	[57]
*EvidenceReport*	The *EvidenceReport* resource is a specialized container for a collection of resources and codable concepts, adapted to support compositions of *Evidence*, *EvidenceVariable*, and *Citation* resources and related concepts.	[66]
*EvidenceVariable*	The *EvidenceVariable* resource describes an element that knowledge (*Evidence*) is about.	[59]

^a^FHIR: Fast Healthcare Interoperability Resources.

^b^EBMonFHIR: Evidence-Based Medicine on Fast Healthcare Interoperability Resources.

^c^HEvKA: Health Evidence Knowledge Accelerator.

### Terminologies

The use of controlled terminologies supports the interoperable representation of multiple concepts. In FHIR, controlled terminologies [68] are represented in code systems and value sets. Reporting a specific term in FHIR uses a *Coding* data type. The *Coding* data type includes a *system* element to identify the terminology system, a *code* element for the precise code for machine use, and a *display* element for human-readable expression. A *CodeableConcept* data type is “a value that is usually supplied by providing a reference to one or more terminologies or ontologies but may also be defined by the provision of text” [56]. A *CodeableConcept* element is composed of a *coding* element (with none, 1, or more instances) and a *text* element (with none or 1 instance).

**Figure 3:**
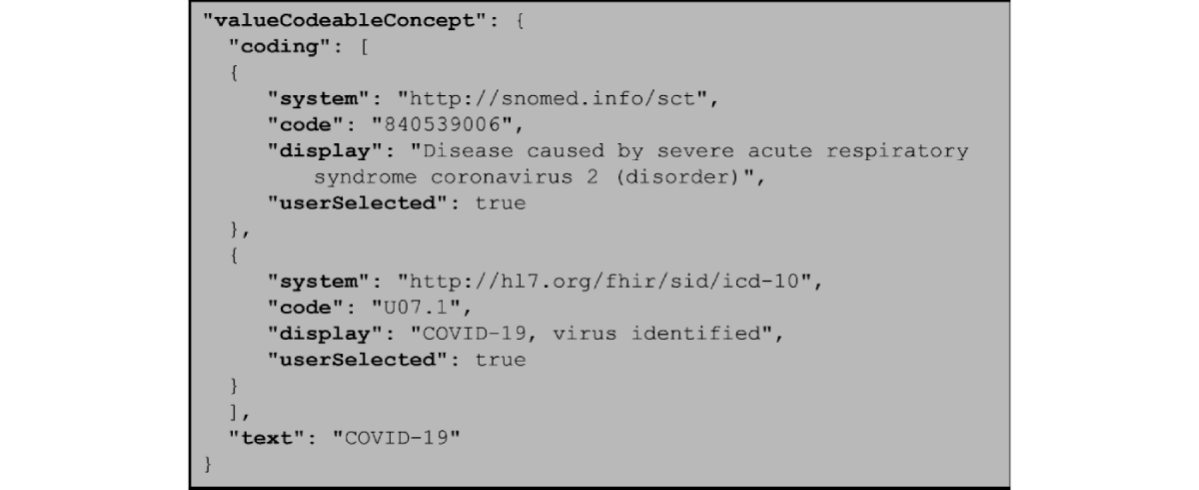
Sample *valueCodeableConcept* element showing multiple *coding* elements. In this example, the *coding* element represents the COVID-19 disease with both SNOMED CT and ICD-10 terminologies. ICD-10: International Classification of Diseases, Tenth Revision; SNOMED CT: Systematized Nomenclature of Medicine—Clinical Terms.

The *coding* element of a *CodeableConcept* element can use a variety of terminologies, such as Systematized Nomenclature of Medicine—Clinical Terms (SNOMED CT), Logical Observation Identifiers Names and Codes (LOINC), RxNORM, and Identification of Medicinal Products (IDMP). FHIR has a noncomprehensive registry of external code systems [68]. New items can be proposed by the community, and implementers can choose to use other code systems not listed in the registry. Figure 3 shows a sample *CodeableConcept* element for the “Disease caused by severe acute respiratory syndrome coronavirus 2.”

HEvKA has used, developed, or extended over 40 terminologies for use with *Evidence*, *EvidenceVariable*, *EvidenceReport*, *Citation*, and *ArtifactAssessment* resources. Consolidation of several of these terminologies has resulted in HEvKA leading the effort to create a Scientific Evidence Code System (SEVCO) with nearly 600 terms for study design, risk of bias, and statistics [50]. Multimedia Appendix 1 presents a list of FHIR data elements with reference to controlled terminologies.

### Walkthrough: Sample Study Result Represented With EBMonFHIR Resources

HEvKA tested the EBMonFHIR standard by conducting a successful proof-of-concept exercise that used the FHIR *Evidence* resource to represent a critically appraised summary of the primary outcome of a multi-platform randomized controlled trial (RCT) of anticoagulation for hospitalized noncritically ill patients with COVID-19 [69]. The primary outcome was “organ support–free days, evaluated on an ordinal scale that combined in-hospital death and the number of days free of cardiovascular or respiratory organ support up to day 21 among patients who survived to hospital discharge” [69]. The primary result for the overall group was reported as a median adjusted odds ratio of 1.27 (95% credible interval 1.03-1.58), with 939 (80.2%) out of 1171 in the therapeutic dose anticoagulation group and 801 (76.4%) out of 1048 in the usual-care thromboprophylaxis group. An overview of FHIR resources used to represent the appraisal of this outcome is shown in Table 2. See the EBMonFHIR walkthrough [70] of this study with details about how the resources are used to represent the study including samples of JSON code to illustrate the EBM representation with FHIR resources.

**Table 2:** List of EBMonFHIR^a^ resources that represent the result for the primary outcome of the sample study.

Reference	FHIR resource type	Description
[71]	*Citation*	Citation for the article in the NEJM^b^
[63]	*Citation*	Citation for evidence resource with FEvIR^c^ Object Identifier (FOI) 7637
[64]	*Evidence*	Summary of 1 unit of evidence (statistical findings for 1 set of variables) from the study
[72]	*Evidence variable*	Exposure in the intervention arm of the study
[73]	*Evidence variable*	Exposure in the comparator arm of the study
[74]	*Evidence variable*	Measured variable for the primary outcome of the study
[75]	*Group*	Group description for the intended population for evidence interpretation
[76]	*Group*	Group description for the observed population in the study

^a^EBMonFHIR: Evidence-Based Medicine on Fast Healthcare Interoperability Resources.

^b^NEJM: New England Journal of Medicine.

^c^FEvIR: Fast Evidence Interoperability Resources.

## Discussion

### Overview

HEvKA is spearheading transformations and advances of EBMonFHIR on several fronts to support the process of exchanging biomedical evidence in a machine-readable format. As a continuously evolving effort, HEvKA has coordinated efforts and collaborated with industry, academia, government, and nonprofit organizations to develop EBMonFHIR resources and related tools, and has presented progress reports and results of the project to both EBM and informatics communities at several national and international events such as American Medical Informatics Association (AMIA) Annual Symposium, American Medical Informatics Association Informatics Summit, Guidelines International Network (GIN) Conference, Mobilizing Computable Biomedical Knowledge (MCBK), and Cochrane Colloquium.

### Status and Maturity of Evidence-Related Standards

The EBMonFHIR standard contains five FHIR resources (ie, *Evidence*, *EvidenceVariable*, *Citation*, *EvidenceReport*, and *ArtifactAssessment* resources) and HEvKA is continually revising and refining the resources with support from members of the EBM community. Currently, the *ArtifactAssessment*, *Citation*, *Evidence*, and *EvidenceVariable* resources are at FHIR Maturity Level 1, “the artifact produces no warnings during the build process and the responsible WG has indicated that they consider the artifact substantially complete and ready for implementation” [77], whereas the *EvidenceReport* resource is at Maturity Level 0 (ie, Draft) and will be deprecated. Dedicated HEvKA working groups are developing functional examples to confirm and demonstrate the use of EBMonFHIR resources and data elements in various use cases. The examples are published on the FEvIR Platform [54]. In addition, following efforts for developing an implementation guide for representing evidence-based CPG recommendations [78], the EBMonFHIR project is producing an EBMonFHIR Implementation Guide [79,80] with 73 profiles of 12 resources to fully represent clinical research and evidence-based guidelines.

### The Role of Controlled Terminologies

Communicating evidence in coded and structured forms requires controlled terminologies (or code systems) to uniquely and accurately express essential concepts. HEvKA created a Code System Development protocol to enable standardized terminologies for the exchange of scientific evidence [50]. This protocol is being executed to develop four code sets related to EBM: study design, statistic type, statistical model, and risk of bias.

Relatedly, the SEVCO Expert Working Group (an offshoot of HEvKA) has 39 members from 18 countries as of November 3, 2023. SEVCO has identified 23 commonly used tools and methods for what the code system will support, such as the ROBINS-I tool for risk of bias assessment [81]. As of this writing, there are 602 prospective terms (253 for risk of bias, 76 for study design, and 273 for statistics) to support all recognized commonly used tools and systems. Coordination with the GRADE Working Group, a collaboration to develop a common and transparent approach to grading quality (or certainty) of evidence and strength of recommendations, is underway to support a GRADE Ontology.

### Tools for End Users

HEvKA participants have developed tools to facilitate the visualization and data entry of evidence into EBMonFHIR resources, including human-readable interfaces and JSON viewers. The tools are integrated into the FEvIR Platform [54], and include intuitive forms, purposefully created to not require manual JSON coding or working knowledge of FHIR, for viewing and building *Citation*, *Evidence*, *EvidenceVariable*, and *Group* resources; for viewing and building multiple profiles of *Composition* resource (*Guideline*, *Recommendation*, *SummaryOfFindings*); for viewing and building multiple profiles of *ArtifactAssessment* resource (*Classification*, *Rating*, *RiskOfBiasAssessment*, and *RecommendationJustification*); and automated converters to translate data from MEDLINE, RIS, ClinicalTrials.gov, and MAGICapp into the FHIR specification. The viewing of resources created with these tools is open without an account, but an account (at no cost) is required to create content.

This environment may reduce the time spent on SR evidence gathering. The laborious process of searching for articles and screening them by hand could be automated because the search query would be expressed in the “language” of EBMonFHIR and judgments made by previous searchers (such as population classification) can be recorded for reuse. Similarly, automated tools could be used to update evidence within SRs rather than relying on manual updates.

With data available in the “language” of EBMonFHIR, it will become easier to develop tools to analyze and process scientific knowledge. Automated meta-analysis tools could be developed based on the recognition of *Evidence* resources with matching *variableDefinition* element content and processing algorithms mapped to the structured *statistic* content. Tools to accelerate original research will also be developed, such as tools to match clinical trials with potentially eligible patients [82].

The *ArtifactAssessment* resource can also be used to represent the quality or certainty of the evidence itself. The EBMonFHIR Implementation Guide includes a *CertaintyOfEvidence* element profile for this purpose. There is also a *DatasetCitation* element Profile of the *Citation* resource, and an *ArtifactAssessment* resource referencing a *DatasetCitation* element can be used to represent the data quality.

### Conclusions

Continuously identifying, synthesizing, and incorporating evidence into care are the key tasks of medical knowledge management, yet they are also prohibitively labor-intensive. HEvKA has taken approaches to standardize and eventually automate these tasks through its efforts around EBMonFHIR, an HL7 standard for making biomedical evidence computable. EBMonFHIR will be used for enabling seamless data flow between published evidence reports, repositories, SR authoring tools, and guideline development tools; automating the searching and matching of evidence with any subgroups of patients; connecting individual patient data with medical knowledge for computerized CDS; and providing individualized effect estimates for different outcomes to facilitate shared decision-making. EBMonFHIR resources in conjunction with other FHIR resources can support relaying EBM components in a manner that is interoperable and consumable by downstream tools and health information systems to support evidence users (eg, creators of biomedical knowledge bases, CPGs, CDS artifacts, and SRs). Anyone may join HEvKA to engage a community of FHIR users and committed volunteers to accelerate the development and implementation of standards for evidence exchange.

## Appendix

Multimedia Appendix 1 List of Fast Healthcare Interoperability Resources (FHIR) data elements with reference to controlled terminologies introduced by the Evidence-Based Medicine on FHIR (EBMonFHIR) project.

## Abbreviations

CDS: clinical decision support
COKA: COVID-19 Knowledge Accelerator
CPG: clinical practice guideline
EBM: evidence-based medicine
EBMonFHIR: Evidence-Based Medicine on Fast Healthcare Interoperability Resources
FEvIR: Fast Evidence Interoperability Resources
FHIR: Fast Healthcare Interoperability Resources
GRADE: Grading of Recommendations Assessment, Development and Evaluation
GIN: Guidelines International Network
GLIF: GuideLine Interchange Format
HEvKA: Health Evidence Knowledge Accelerator
HL7: Health Level Seven
IDMP: Identification of Medicinal Products
LOINC: Logical Observation Identifiers Names and Codes
MCBK: Mobilizing Computable Biomedical Knowledge
RCT: randomized controlled trial
SAGE: Standards-Based Sharable Active Guideline Environment
SEVCO: Scientific Evidence Code System
SNOMED CT: Systematized Nomenclature of Medicine—Clinical Terms
SR: systematic review
UML: unified modeling language
