# Supplementary material for: Making Science Computable Using Evidence-Based Medicine on Fast Healthcare Interoperability Resources: Standards Development Project
Source: J Med Internet Res. 2024 Jun 25;26:e54265. doi: 10.2196/54265 (PMC11234056; doi:10.2196/54265)
Supplement: Multimedia Appendix 1 [file jmir_v26i1e54265_app1.docx]

List of FHIR data elements with reference to controlled terminologies introduced by the EBMonFHIR project (as of November 2, 2023).

| **Resource** | **Element Path** | **Binding Strength** | **Value Set** |
| --- | --- | --- | --- |
| Evidence | .variableDefinitition.variableRole | Extensible | variable-role |
|  | .variableDefinitition.directnessMatch | Extensible | directness |
|  | .synthesisType | Extensible | synthesis-type |
|  | .studyDesign | Extensible | study-design |
|  | .statistic.statisticType | Extensible | statistic-type |
|  | .statistic.attributeEstimate.type | Extensible | attribute-estimate-type |
|  | .statistic.modelCharacteristic.code | Extensible | statistic-model-code |
|  | .statistic.modelCharacteristic.variable.handling | Required | variable-handling |
|  | .certainty.type | Extensible | certainty-type |
|  | .certainty.rating | Extensible | certainty-rating |
| Evidence  Variable | .definition | Example | evidence-variable-definition |
|  | .handling | Required | variable-handling |
| Evidence  Report | .type | Example | evidence-report-type |
|  | .subject.characteristic.code | Extensible | focus-characteristic-code |
|  | .relatesTo.code | Required | report-relation-type |
|  | .section.focus | Extensible | evidence-report-section |
|  | .section.entryClassifier | Extensible | evidence-classifier-code |
| Citation | .summary.style | Extensible | citation-summary-style |
|  | .classification.type | Extensible | citation-classification-type |
|  | .classification.classifier | Example | citation-artifact-classifier |
|  | .currentState | Example | citation-status-type |
|  | .statusDate.activity | Example | citation-status-type |
|  | .citedArtifact.currentState | Extensible | cited-artifact-status-type |
|  | .citedArtifact.statusDate.activity | Extensible | cited-artifact-status-type |
|  | .citedArtifact.title.type | Extensible | title-type |
|  | .citedArtifact.abstract.type | Extensible | cited-artifact-abstract-type |
|  | .citedArtifact.part.type | Extensible | cited-artifact-part-type |
|  | .citedArtifact.relatesTo.type | Required | related-artifact-type-all |
|  | .citedArtifact.relatesTo.classifier | Extensible | citation-artifact-classifier |
|  | .citedArtifact.publicationForm.publishedIn.type | Extensible | published-in-type |
|  | .citedArtifact.publicationForm.citedMedium | Extensible | cited-medium |
|  | .citedArtifact.webLocation.classifier | Extensible | artifact-url-classifier |
|  | .citedArtifact.classification.type | Extensible | cited-artifact-classification-type |
|  | .citedArtifact.classification.classifier | Example | citation-artifact-classifier |
|  | .citedArtifact.contributorship.entry.contributionType | Extensible | artifact-contribution-type |
|  | .citedArtifact.contributorship.entry.role | Extensible | contributor-role |
|  | .citedArtifact.contributorship.entry.contributionInstance.type | Extensible | artifact-contribution-instance-type |
|  | .citedArtifact.contributorship.summary.type | Extensible | contributor-summary-type |
|  | .citedArtifact.contributorship.summary.style | Extensible | contributor-summary-style |
|  | .citedArtifact.contributorship.summary.source | Extensible | contributor-summary-source |
| Artifact  Assessment | .content.informationType | Required | artifactassessment-information-type |
|  | .content.type | Example | certainty-type |
|  | .content.classifier | Example | certainty-rating |
|  | .workflowStatus | Required | artifactassessment-workflow-status |
|  | .disposition | Required | artifactassessment-disposition |
